# Patients’ perspectives on screening for disordered eating among adolescents with type 1 diabetes

**DOI:** 10.1007/s40519-023-01539-2

**Published:** 2023-02-08

**Authors:** Caroline Bruun Abild, Annesofie Lunde Jensen, Rikke Bjerre Lassen, Esben Thyssen Vestergaard, Jens Meldgaard Bruun, Kurt Kristensen, Rene Klinkby Støving, Loa Clausen

**Affiliations:** 1 Steno Diabetes Center Aarhus, Aarhus University Hospital, Aarhus, Denmark; 2 Department of Clinical Medicine, Aarhus University, Aarhus, Denmark; 3 Pediatric Clinic, Regional Hospital Randers, Randers, Denmark; 4 Center for Eating Disorders, Odense University Hospital, Odense, Denmark; 5 Research Unit for Medical Endocrinology, Odense University Hospital, Odense, Denmark; 6 Mental Health Services in the Region of Southern Denmark, Esbjerg, Denmark; 7 Department of Child and Adolescent Psychiatry–Research unit, Aarhus University Hospital, Aarhus, Denmark; 8 Danish National Center for Obesity, Copenhagen, Denmark

**Keywords:** Adolescent, Pediatric, Eating disorder, Eating disorder behavior, Diabetes, Screening tool

## Abstract

**Purpose:**

People with type 1 diabetes have an increased risk of disordered eating (DE) and eating disorders (ED). Screening is recommended however little is known about patients’ perspectives on screening questionnaires. This paper reports qualitative analyses of patients’ perspectives on the questionnaire Diabetes Eating Problem Survey Revised (DEPS-R), including acceptability, attitudes, and cognitive understanding.

**Research design and methods:**

15 adolescents with type 1 diabetes between 11 and 18 years, were interviewed. A semi-structured format and a qualitative Interpretive Descriptive (ID) methodology was chosen.

**Results:**

The analyses identified four themes: (1) The Questionnaire, (2) Reframing Diabetes Visits, (3) This is (not) for me, and (4) Out in the Open. The DEPS-R was completed with-in 5–10 min. with no technical difficulties. The questionnaire altered the diabetes visit for some, creating a new dialog, and time for self-reflection. Adolescents appreciated the direct approach in the questionnaire, and showed willingness to complete the questionnaire, when presented to them by a health care professional (HCP). One item in the DEPS-R proved difficult to understand for some participants.

**Conclusion:**

The study highlights DEPS-R as a clinically relevant screening questionnaire. Completing DEPS-R prior to a consultation opens the door to a consultation that invites the adolescent to address matters of eating behavior. Our findings suggest that systematic screening of DE/ED using the DEPS-R is both accepted and welcomed by adolescents with type 1 diabetes. Future research should focus on a potential update of selected items in DEPS-R.

**Level of evidence:**

V – qualitative study.

## Introduction

Type 1 diabetes is a chronic auto-immune disease characterized by deterioration of insulin-producing beta cells in the pancreas, leading to insulin depletion and hyperglycemia [1]. Individuals with type 1 diabetes have a 2–threefold increased risk of disordered eating (DE) or eating disorders (ED) [2–7]. Coexisting type 1 diabetes and DE/ED is associated with impaired metabolic control and consequently an increased morbidity and mortality [8–10].

The etiology behind DE/ED in type 1 diabetes is unknown however contributing factors may be insulin-dependent weight (re)gain after diagnosis, incessant glucose monitoring and subsequent insulin dosage, adding dietary restraint and preoccupation as part of the diabetes management [11–13].

ED refers to clinical EDs meeting diagnostic criteria for anorexia nervosa, bulimia nervosa or binge eating disorder[14], whereas DE refers to inappropriate eating behaviors and preoccupation with weight, body shape or eating, however, not yet at a level, frequency, or severity to qualify for a formal ED diagnosis [13]. Insulin manipulation or omission is a diabetes specific weight loss technique that is uniquely applicable to patients with type 1 diabetes. Up to 40% of young women with type 1 diabetes report insulin omission with the purpose of weight loss [10, 15].

Due to this poor prognosis guidelines recommend routinely screening for DE/ED in type 1 diabetes [16, 17], as early intervention may improve the outcome, especially in children and adolescents [18–20]. One of the fundamental principles of screening is that a test should be acceptable to the population [21]. This has to our knowledge never been investigated regarding DE/ED screening in adolescents with type 1 diabetes. Numerous screening tools are available to detect symptoms of DE/ED. However, generic screening tools may estimate the prevalence inaccurately in people living with diabetes, due to the required focus on nutrition, weight, and carbohydrate intake. Furthermore, these tools do not include the aspect of insulin manipulation [3, 22]. Diabetes Eating Problem Survey—Revised (DEPS-R) is a 16 items screening tool to detect DE in diabetes [23]. Higher scores indicate greater pathology, and a score ≥ 20 Likert point is positively correlated with signs of DE [23, 24]. Although reliability and validity of DEPS-R have been extensively tested with acceptable results [25–27], the patients’ perspectives on DEPS-R have never been explored.

The aim of this study was to investigate perspectives of adolescents with type 1 diabetes on systematic screening using DEPS-R prior to a yearly visit. This includes their cognitive understanding, experiences completing the questionnaire, as well as their acceptance of time consumption, question content, and utility in the clinical consultation.

## Methods

### Study design

An explorative, qualitative research approach using individual semi-structured interviews was chosen to explore perspectives, convey attitudes, acceptability, and cognitive understanding of the Danish translation of DEPS-R among adolescents with Type 1 Diabetes [28, 29]. Most answered DEPS-R at home before the visit, expressing that it “was easier to concentrate”, but one filled out the questionnaire in the car going to the clinic.

Interpretive Description (ID) served as the methodological framework both regarding design and interpretation [29]. A relevant key element of ID is the importance of clinical relevance and application matching our aim derived from a clinical perspective and intending to improve and inform clinical practice.

To ensure both conduct and reporting of the study the CoreQ 32-item checklist was introduced [30].

### Participants

Participants were recruited from the Pediatric and Adolescents outpatient Clinic at Steno Diabetes Center Aarhus (SDCA). Participants were recruited using purposive sampling [29]. Potentially eligible participants were identified through medical records of scheduled appointments at the clinic, using the following inclusion criteria (1) Type 1 diabetes duration > 1 year, (2) between 11 and 18 years old, and (3) completed the DEPS-R questionnaire ≥ 2 times. Patients were approached by their health care professional (HCP). The sampling strategy targeted heterogeneity in age, gender, and DEPS-R score. The sample size was chosen inductively reliant on the concept on “information power” rather than “data saturation” [31]. Information power, and therefor sample size, is among others dependent on the aim of the study, sample specificity, and quality of dialog. The aim of this study is rather broad in terms of capturing participants perspectives pointing toward a larger number of participants (e.g., *N* = 20), however, the sample specificity is indeed targeted due to patient group, and the interviewers experience with patient group elicits strong and clear communication, pointing toward a smaller sample size (e.g., *N* = 10).

### Data collection

Semi-structured interviews were performed during winter 2020 until fall 2021 by first (CBA) and third (RBL) author, both experienced interviewers, neither have met the participants before, which enabled the participants to freely express opinions in relation to treatment and the clinic. The interviews were performed either by telephone, online or in-person, following Covid-restrictions at the time.

To explore acceptability of implementing a systematic screening using DEPS-R among participants, specific questions regarding technicalities and time consumptions were addressed in the interviews.

Some HCPs reported curiosity regarding cognitive understanding, but also concerns regarding potential suggestiveness of inappropriate behavior in relation to specific questions in the DEPS-R. To explore and include these considerations, CBA participated in several Multi-Disciplinary Team (MDT) meetings at SDCA, and in collaboration with HCP four items were selected; (3) “Other people have told me that my eating is out of control”, (4) “After I overeat, I don’t take enough insulin to cover the food”, (7) “I avoid checking my blood sugar when I feel like it is out of range”, (10) “I try to eat to the point of spilling ketones in my urine”. To explore comprehension of these individual items, a cognitive interviewing technique was applied [32, 33]. During the interviews the participants were asked to rephrase and comment on these selected questions. It has been proposed that item 10 could give rise to inappropriate ideas among susceptible adolescents [34]. Hence, the origin of the knowledge of spilling ketones was explored specifically.

### Data analysis

The interviews were transcribed verbatim and read numerous times to establish familiarity. Qualitative analysis was performed using an iterative and inductive thematic approach in accordance with the ID methodology. An apprehensive approach, in relation to premature coding of themes, ensured the inductive cycle [29].

The initial coding was performed by the first author in Nvivo Software, systemizing transcripts, and themes. The emergent themes were then discussed by parts of the author team, including terms, interpretation, relationship between themes and clinical relevance and implications. Citations in the present manuscript were translated from Danish by the authors.

### Ethical considerations

All participants were provided written information about the study and a consent formula in preparation to participation in interview. The consent included a parental consent for those ≤ 15 years. All participants were given a window of reflection to consider participation. All data were handled in accordance with General Data Protection Regulation (GDPR).

The study was registered at The Science and legal office, Central Denmark Regional Office (case number 1-45-70-21-21). The Committee on Health research Ethics evaluated the project as not needing additional specific approval.

## Results

**Fig. 1:**
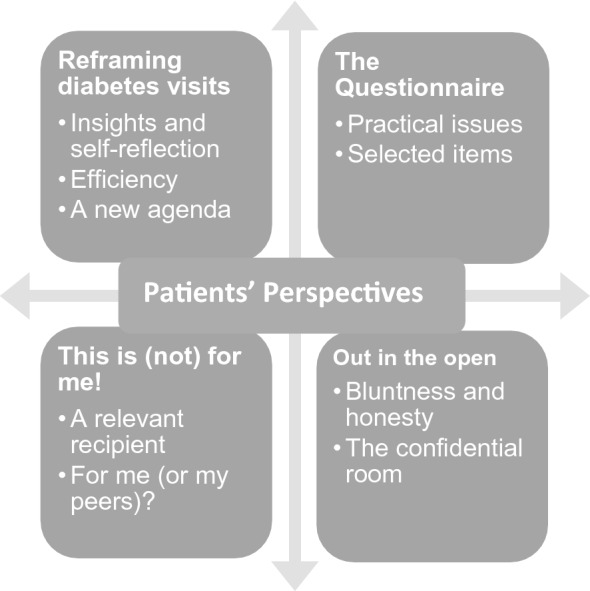
Themes and sub-themes derived from the analysis of interviews exploring patients’ perspectives on screening for disordered eating in type 1 diabetes

15 participants with type 1 diabetes between the age of 11–18 years were included, with a mean age of 15.1 (± 1.8) years. There were no differences in age among sexes. Participants had a mean DEPS-R of 10.8 (± 11.3), revealing an expected higher DEPS-R score among females 15.5 (± 12.4) than males 5.4 (± 7.5). Time consumption per questionnaire was 7.6 (± 4.1) minutes (Fig. 1, Table 1).

**Table 1:** Participant characteristics and DEPS-R score

	All *N* = 15	Females *N* = 8	Males *N* = 7
Age (years)	15.1 (± 1.8)	15.3 (± 1.3)	15.0 (± 2.4)
11–14	7	12.7 (± 1.5)	13.8 (± 0.5)
15–18	8	16.4 (± 1.1)	16.3 (± 1.2)
DEPS-R score (points)	10.8 (± 3.2)	15.5 (± 4.7)	5.4 (± 3.4)
0–19	73.3%	7.2 (± 6.9)	2.2 (± 3.0)
≥ 20	26.7%	29.3 (± 7.8)	25.0 (± 0.0)
Time consumed per questionnaire (minutes)	7.6 (± 4.1)	8.6 (± 4.9)	6.4 (± 2.8)

Data are mean and standard deviation scores in parenthesis

*DEPS-R* Diabetes eating problem survey revised, *F* females, *M* males

The analysis revealed 4 themes, each theme containing 2–3 subthemes.

### The questionnaire

The first theme includes two subthemes: *Practical issues* and *Selected items*. The theme reflects findings related to practicalities regarding completion of the questionnaire and the understanding of selected items in the DEPS-R.

Regarding *Practical issues*, all participants completed the questionnaire online, and none reported technical difficulties accessing the link. Most participants filled out the DEPS-R on their cell phone, however some by computer. Some reported that their parent opened the questionnaire and then called them, but most of them accessed the link themselves after having received a reminder by a text message. All access to the questionnaire required a two-factor authentication.

Participants reported the time consumption being acceptable with responses between 5 and 15 min. Their subjective sense of time consumed, was in accordance with real time spend on the questionnaire, when compared to data in the electronic patient journal.

*Selected items* in DEPS-R were investigated. Item 3, “Other people have told me that my eating is out of control”, was investigated to explore how the participants interpreted the “out of control” phrasing. Most participants understood the question, but some found it irrelevant, which made it difficult to answer.

Several participants made it clear, that eating habits were inevitably coined to diabetes and diabetes treatment.

*“If you go and snack a lot without taking insulin to cover it. It could be, that you get hungry, and instead of eating a proper meal, you go snacking for an hour. That I would say. Out of control.”*

Hence, ‘out of control’ was more likely linked to insulin omission, resulting in an understanding of ‘loss of control over the diabetes’, more than the eating behavior.

Item 4, “After I overeat, I don’t take enough insulin to cover the food”, address the complex concept of overeating, as well as purposely omitting insulin. Participants are all aware that inadequate insulin dosage, voluntarily or involuntarily, results in hyperglycemia. For all participants, the question posed no real difficulties in understanding at first glance:

*” If you eat a lot, you don’t give enough* [insulin]*.”*

Nevertheless, the extend of overeating in a pathological way, as objectively larger quantities of food, with loss of control, does not seem to be adequately understood.

Several participants continued to link eating behavior, both appropriate and inappropriate, to diabetes management, signaling that it is difficult to address eating, without automatically linking to diabetes compliance. The participants understood overeating as extra food eaten, beyond the first or planned portion, covered by estimated insulin dosage.

Several participants linked overeating to a specific macronutrient, namely carbohydrates, thereby continuously underlining the connection to the diabetes:

*“I have eaten more carbohydrates than the carbohydrates I have taken insulin to cover. I think mostly of overeating as overeating of carbohydrates, but maybe it’s not like that for everyone. But it’s like that for me.”*

For some participants, all scoring above cut-off in the DEPS-R, the second portion seemed more manageable if not registered in the insulin pump, thus creating less shame. Some implied, that ‘just taking all your insulin’, especially if it is a large amount, was equivalent to ‘doing something wrong’. The number of units of insulin indicates the amounts of ingested carbohydrates, therefore a larger than normal insulin dose, felt like confirmation of overeating for some participants. Summing up, overeating was by adolescents with type 1 diabetes generally not comparable to the definition of bulimic binge eating.

Item 7, “I avoid checking my blood sugar when I feel like it is out of range”, was easily comprehensible to all.

*“When you don’t check your blood sugar, when you feel that it is outside of the range, where it is supposed to be. That is, you avoid checking it, if it’s too high or too low*.”

Almost all participants flatly responded, “that’s between 4 and 8 mmol/l” reflecting a clear understanding of what is outside the range.

Regarding item 10, “I try to eat to the point of spilling ketones in my urine”, some HCP worried that it was too complex for patients to answer or that it might give adolescents inappropriate ideas. Approximately one-third of participants had difficulties understanding this question. Some participants explained this correctly but felt very insecure and were therefore categorized as non-comprehensive. The level of understanding was independent of age and sex. The participants in the non-comprehensive group ranged from age 13–17 year, compared to the comprehensive group ranged from age 11–18 year. There was an equal number of female and males in both groups. It was on the other hand somehow dependent of DEPS-R score as those in the non-comprehensive group had a lower DEPS-R score than the comprehensive group. Some of those with difficulties understanding the question, expressed that an introduction before the first time completing the questionnaire could be helpful.

The remaining participants have a comprehensive understanding:

*” It means eating so many carbohydrates, or not taking insulin for what you eat, that you spill out ketones in your urine. I don’t think that way. I just think that my blood sugar gets so high that I’ll lose weight.”*

This participant was clearly aware of the physiological mechanisms and linked the behavior to weight loss. Participants expressed that the origin of the knowledge stems from diabetes education at the time of diagnosis, private surroundings, and social media. None of the participants expressed that the questionnaire provided incentive for inappropriate behavior.

### Reframing diabetes visits

Participants expressed that the questionnaire reframed the diabetes visit as it contributed to increased *Insights and self-reflection,* made the visit more *Efficient*, and created *A new agenda.* This theme reflects the changes that the questionnaire entailed at the diabetes visit.

The subtheme *Insights and self-reflection* revealed that participants reached a new view on their situation when completing the questionnaire. It was described as a short break from an everyday life with diabetes, enabling them to ‘stop up’ and think about ‘how they are really doing’. This enabled them to broach new subjects:

*Well, I have clearly realized some things by answering them. But it is not necessarily bad I would say. I.e., this “if I have overeaten, I haven’t taken enough insulin for the food I have eaten”. When I answer that, I think “Oh my, that’s really stupid. Like, why do you do it?”*’

Overall, participants expressed that completing the questionnaire created a reflection room for them and contributed to both personal and disease related insights.

All participants were aware of the time limit at a diabetes visit and maybe therefore felt positive about the *Efficiency*, the questionnaire entailed at a diabetes visit, as being a preparatory task, optimizing the time at the diabetes visit to vocalize possible needs at the diabetes visit.

The positive approach was especially dominant among those scoring below cut-off on the DEPS-R because they were reluctant to spend valuable clinical time on perceived irrelevant issues.

*“I think maybe people are more honest and have more time to think about the answers when they sit at home and answers. Also, you don’t have ‘all the time in the world’ at a visit, so maybe it’s a way of saving time”.*

Participants indicated that the efficiency interacted with both honesty and the possibility of increased self-reflection prior to the diabetes visit.

All participants agreed on the existence of an unspoken agenda at diabetes visits, focusing on “the highs and the lows”. Some participants expressed that the questionnaire enabled *A new agenda* at the diabetes visits:

*“Yes, like before, they just looked at your blood sugars, how it was and like that, and then maybe they started asking ‘how come you have so many highs or lows’, and then you must be truthful. But then you can say, that in the food and body [*part of questionnaire]*, they can start to ask, like if you answer always on the urine, then they can say, ‘why aren’t you taking your insulin, is it to lose weight or what?’”*

The diabetes visits changed, from being “just about the diabetes” to reflecting the circumstances surrounding the adolescent. The agenda and dialog changed, especially among those with a DEPS-R score ≥ 20 the agenda changed at the subsequent visits, because of the possibility of evaluating changes in DE and general well-being.

### This is (not) for me!

This theme consists of the subthemes *A relevant recipient* and *For me (or my peers)?* exploring the participants’ feelings of (ir)relevance toward the DEPS-R.

The feeling of being a *Relevant recipient*, was related to, and dependent on, the level of information delivered from the HCP prior to the first questionnaire.

*“In a way you feel safer, if your doctor says it, because then you know it’s legit. It’s not just something you’re offered. You sometimes get questionnaire from all over places. When your doctor tells you, it feels safer.”*

All participants conveyed a feeling of security and increased importance, when the questionnaire was introduced to them by a known HCP, at least the first time.

Some participants experienced a sense of irrelevance; is this relevant *For me (or my peers)?* However, at the same time the participants expressed ambivalence on this subject, as they felt part of a diabetes community, indicating a willingness to complete a questionnaire because peers may feel this way.

*“For instance, there’s the question about ketones. Because I have never thought about it, but others do. So, I understand that it’s included.”*

Even though not individually relevant, there was a collective understanding; “us with diabetes”, with different needs.

Summing up, the sense of relevance when completing a questionnaire was essential to compliance among participants. The sense of relevance was highly influenced by an introduction to the questionnaire, including content and incentive, by a known HCP.

### Out in the open

This theme consists of two subthemes, *Bluntness and honesty*, and *The confidential room* reflecting two different aspects of the open communication in relation to the questionnaire.

When including a questionnaire on DE in the clinic, the silent becomes declared. The sub-theme *Bluntness and honesty* reflect how participants' thoughts are vocalized and how DE as an existing phenomenon gets endorsed:

*“I think it is a form of recognizability. It’s not something completely unfamiliar to you either. I don’t know. It’s something, a lot (*of people*) know about. It’s not a taboo in the same way.”*

Other participants elaborate on the shared knowledge among them and HCP:

*“I hadn’t tried it before (*answering the questionnaire*), but in a way it was quite good, that the focus shifted. It was nice, that the questions were so blunt. I think, that regarding food and insulin restrictions, a lot know about this, and we haven’t talked about it before. So, the questionnaire shows, that you know that we know. It’s not a secret.”*

The systematic screening or awareness of warning signs for DE/ED was a novel thing, however, the bluntness of bringing these issues out in the open signaled that they were not considered a taboo which was welcomed by participants.

All the participants involved their parents to some extent. About half of the participants discussed the questionnaire with their parents, while completing it. Some because they had difficulties understanding the questions, others because they acknowledge symptoms of DE, and again some because diabetes and its management, is a common issue for the family. Others completed the questionnaire themselves, and the parents were involved rather abruptly at the consultation, perhaps creating an unwanted leak in the *Confidential room*:

*“I always think that it’s a bit uncomfortable. I must admit. I feel like the questionnaire is mostly for the doctors and me, and it’s about the secret message. It’s something between me and the doctors. When my parents are present, and hears it, I get like “ups”. Sometimes it’s a bit uncomfortable I must admit.”*

## Discussion

The present study is the first to explore patients’ perspectives on screening for DE/ED in type 1 diabetes and shows that DEPS-R is both clinically relevant and acceptable among adolescents living with type 1 diabetes.

We found an overall acceptable understanding of the DEPS-R, however selected items were difficult to understand for some. In item 3, “Other people have told me that my eating is out of control”, some misconstrued the question as being entangled with diabetes compliance, as is also the case with item 4, “After I overeat, I don’t take enough insulin to cover the food”. Awareness of level of cognitive understanding is needed, as overeating with loss of control as pathological bulimic binge eating was misconstrued by most participants. I.e., some patients understood overeating as eating more than planned or snacking without adequate insulin dosages as opposite to the definition of pathological binge eating including objectively large amounts eaten within a short duration of time, with loss of control, followed by shame and self-loathing. It is common in questionnaire surveys, both among children, adolescents, and adults to report more binge eating than when clinically assessed, probably because of the very specific definition of pathological overeating not reflecting a subjective understanding [35]. Additionally, overeating and binge eating during episodes of hypoglycemia are common and can be associated with either ED pathology or other more diabetes specific causes, however, no adolescents reported episodes like this. Future studies should explore whether item four should be interpreted with caution as a high score of binge-eating symptoms could be related to episodes of hypoglycemia more that ED pathology. Item 7, “I avoid checking my blood sugar when I feel like it is out of range” is another item needing caution as it reflects noncompliance in diabetes care which can have many different reasons not necessarily related to eating problems. However, for those with ED or DE it can indicate purposeful and inadequate response to correct a high or low blood glucose.

Item 10, regarding ketones, challenged a third of participants. Correspondingly, this question has been omitted in the Dutch version of DEPS-R [36]. As the majority understand the item, we suggest further research before omitting this question in general or rephrasing it. However, a preliminary introduction by an HCP is important regarding comprehension and sense of relevance. Some HCP, as well as researchers, have prompted discussion on the potential suggestive behavior a questionnaire on DE could have [34], but our study does not support this. This knowledge should be disseminated to avoid future over-concern regarding implementing similar screening procedures. The findings on selected items should entail further investigation and possible adjustment of the item to ensure the validity of the questionnaire.

Completing DEPS-R prior to a consultation yields possibilities for a new and different diabetes visit, but also for personal insights and self-reflection. The questionnaire entailed time for preparation and gave rise to discussion among families of most essential topics to address at the visit. This is important for clinicians to acknowledge, to accept and to accommodate accordingly. Besides potential personal insights, the use of DEPS-R as a screening tool, could prompt important disease insights and give incentive to more active participation in treatment. This how found to be associated with improved health outcomes in terms of metabolic regulation and quality of life [37].

Screening for disordered eating and eating disorders can however be unchartered territories for some HCP, being apprehensive in broaching the sensitive topic with their patients [38]. Research shows that HCP have reported difficulties managing the care of individuals with comorbid ED and T1D due to a need for more knowledge [38]. Hence, implementing systematic DE/ED screening should include training on DE/ED for diabetes HCP.

The results highlight the importance of HCP respecting adolescent confidentiality in the screening process. Awareness of the independency and need for both a confidential room and the possible assistance of parents must be balanced both in the process of completing the questionnaire and in the subsequent feedback by the HCP as a natural transition into adulthood. Studies both report the significance of maintaining a productive communication among adolescents with type 1 diabetes and their parents in phases of rising autonomy [39–41] and that the degree of autonomy plays a significant role in the self-care in adolescents with diabetes [40, 42, 43]. Thus, HCP should encourage parental communication strategies that enhance autonomy development among adolescents. This approach could be supported in segregating the diabetes visits into individual and family consultations among selected patients.

In the study we included participants from 11 to 18 years old. Research shows that DE including insulin omission increases with age from pre-teen to early adolescence as only 1% of pre-teen girls have tried to omit insulin, whereas > 30% will have experienced this behavior in late teens and early adulthood [44]. This points toward an extension of the screening procedure to including young adults. This is also supported in the average score of DEPS-R peaking in late teenage and early adulthood [45].

### Strengths and limitations

The semi-structured interviews provided rich and novel data regarding patients’ perspectives on completing DEPS-R in a clinical setting. However, a few limitations must be addressed when drawing conclusions. Participants were initially identified by researchers but approached by a well-known HCP with special knowledge about the person, thereby potentially creating a selection bias where the HCP acted as a gatekeeper. Gatekeeping is a common phenomenon within health care research, as they do have responsibility to protect potentially vulnerable people [46]. This recruitment could have skewed the elected study population, favoring those with a high functioning level. The sample is representative of the general adolescent population with T1D regarding the smaller proportion of participants with a cut-off ≥ 20 DEPS-R. However, the analysis could have benefitted from additional perspectives stemming from a larger sample of participants scoring above cut-off as differences in attitudes and cognitive understanding are somewhat dependent on DEPS-R score.

The inclusion criteria “completion of the DEPS-R questionnaire ≥ 2 times” could cause another selection bias as patients with challenges in their completion were excluded. Another potential selection bias is the investigation of selected items in the DEPS-R. The items were selected in collaboration with clinicians in the project, as the items causing the most frustration, discussion and misunderstanding among patients. Retrospectively, we could have considered selected items with patients in the interview setting or conducted a focus group interview. Lastly, as this study applies an ID methodology and focuses on individual experiences and insights, some results may not be transferrable to other settings.

## What is already known on this subject?

The prevalence of ED/DE among people living with type 1 diabetes is elevated and international guidelines recommend screening for ED/DE, however, to the best of our knowledge patients’ perspectives on completing DEPS-R in a clinical setting have not yet been investigated.

## What this study adds?

Several clinical and research implications can be drawn from this study. Our study contributes to the understanding of screening for DE/ED with a diabetes specific questionnaire, yielding great prospects for an early detection of DE symptoms by facilitating an open dialog and potential aid for those with DE. The study also highlights the importance of information on DEPS-R, including the incentive and content of the questionnaire, to increase a sense of relevance and security. Using the DEPS-R is both accepted and welcomed by adolescents with type 1 diabetes. Future research should focus on rephrasing or updating selected items, and investigating clinicians’ perspectives on implementing a systematic screening for DE/ED using DEPS-R.

## Data Availability

Data is available on demand.
